# Book Review

**Published:** 1905-09

**Authors:** 


**The Medical Annual for 1905.** Pp. lxxxiv., 704. Illustrated. Bristol: John Wright & Co.—This volume commences a new series, in that the page has been increased in length to correspond with the increasing thickness of the annual issues. The editor in his preface says, "We have tried to do our work so that no page of the *Annual* shall be without interest and information to the practitioner," and we can state, as one of the readers, that the editor has always succeeded admirably in this respect. Though the use of cross references has rendered it unnecessary, we should have been glad to see the new series grouping the various diseases more completely under the larger headings of diseases of the lungs, nose, skin, &c., respectively, or, if the alphabetical arrangement of diseases as headings is to be retained, that a scheme of the various pulmonary, nose, skin, &c., complaints should be given somewhere, so that anyone wishing to see at a glance the advances reported under these several headings could do so without wading through the whole work or index. This, however, is a suggestion, not a complaint, for it is difficult to criticise when the whole is so excellent.

We note, however, that an article on the Nordrach treatment of consumption, written by Dr. Penn-Milton, is attributed to Dr. Walther, an inaccuracy which should have been avoided.

We must especially congratulate the editor and the publishers on the series of stereograms, showing that the difficulties in the printing of these plates have been overcome in a most creditable manner; we have never seen finer examples of the printer's art in any text-book, while the stereoscope supplied by the publishers, an improved modification of Watson Williams's stereoscope, is the best hitherto produced for stereograms in a book.

**Serums, Vaccines and Toxines.** By Wm. Cecil Bosanquet, M.A., M.D. Pp. viii., 344. London: Cassell & Co. Limited. 1904.—This, a small and concise work on a very difficult subject, is a book which was very much wanted by the medical profession. The theories are put as shortly and clearly as possible, and their practical aspect explained. There is just sufficient history to add an interest to the subject. The chapters on Diphtheria and Typhoid are particularly good. The treatment of rabies by Pasteur is explained and approved, and the few statistical tables on this subject are carefully chosen and present a most convincing statement. Hæmolysis and cytolysis, subjects not easy to understand in most descriptions, can be understood without difficulty. The same may be said for precipitins and agglutinins. The book does not lay any claim to originality, but great credit must be given to the author for sifting the tangled maze of terms which have been introduced into this subject and presenting them in a clear, readable form, in a small compass, and in a way that the busy practioner can follow without the use of a dictionary.

**Medical Electricity.** By H. Lewis Jones, M.A., M.D. Fourth Edition. Pp. xvi., 536. London: H. K. Lewis. 1904.—This edition of Lewis Jones's valuable text-book on things electrical in medical practice contains additional chapters on the following subjects: The Utilisation of Currents from the Mains for Medical and Surgical Purposes, High Frequency Electricity, and also an introduction to the work with X-ray machines. The subject of the use of electricity in practical medicine has made and is making such rapid progress that it is difficult for writers to keep their text-books up to date. This work is of necessity incomplete, but it is a book ready of reference, well arranged, and unbiased in opinion on controversial topics. If it is difficult to keep text-books up to date, how much harder is it for the practitioner to keep pace with the development of this side of practical physics unless, indeed, he make this branch a special study! A volume such as this is serves to show how important the knowledge of physics may prove in the education for the profession. There is, indeed, it appears, no subject of which a medical man may feel that the knowledge is of no importance. The treatment of disease by physical methods other than electricity has also a wide scope, and the future student will be required to show a grasp of these things before he can be said to be equipped with modern weapons.

**Walsham's Handbook of Surgical Pathology.** Third Edition. Revised by Herbert J. Paterson, M.A., M.B. Pp. xxii., 529. London: Baillière, Tindall & Cox. 1904.—When the first edition of this work came out in 1878 it was quite a new idea in this country. Its great use was at once felt by the students of St. Bartholomew's Hospital: it couples up their works on Surgery and Pathology with the actual specimens in the museum. The subject is described in the text, and then certain typical specimens are referred to and explained. The present edition has been much enlarged, and cases in the College of Surgeons are also used for reference. The whole work is excellently got up, well printed, and is very pleasant reading. Even apart from reading in the museums, to which it specially refers, the book gives an excellent survey of surgical pathology.

**The Principles and Practice of Asepsis.** By Arthur Styles Vallack, M.B., Ch.M. Pp. x., 95. London: Baillière, Tindall and Cox. 1905.—This little book is a reliable and succinct account of the lines on which the surgical or obstetric operator should work in attempting to secure asepsis. He gives in detail the precautions which must be taken in order to prevent infection of wounds. Many of these are common knowledge at the present time, but the valuable feature of this book is that it provides in a small space a combination of all the information on the subject, rendering reference to many isolated papers by different authors unnecessary to those in search of information on various points of detail. He shows that infection from airborne germs is not of the frequency or importance once attributed to it, while infection from the skin of the patient or operator is both common and virulent. Following Kocher and Leedham-Green, he shows the great difficulty of rendering the skin of the hands aseptic, and is a strong advocate for the wearing of gloves by the surgeon and the isolation of the area of operation by the use of sterilised sheets stitched to the edges of the wound. We notice that he does not allude to the infection of wounds by the saliva or breath of the operator, and also an obvious misprint on page 74, the sentence, "If septic, shut up; if aseptic, keep open," obviously should be, "If *aseptic*, shut up; if *septic*, leave open."

**A Handbook of Intestinal Surgery.** By Leonard A. Bidwell. Pp. xi., 167. London: Baillière, Tindall, and Cox. 1905.—This little book is mainly intended for the help of those performing operations on the dead subject, and contains a full description of the work done in intestinal surgery in the class at the West London Post-graduate College. From this point of view it is a useful handbook, as the various procedures are lucidly explained and the text is fully illustrated with figures which are largely semi-diagrammatic. The methods are, however, such as are common knowledge to the operating surgeon, and can be found in the larger manuals dealing with gastro-intestinal operations.

**Recurrent Effusion in the Knee-joint.** By Sir William Bennett, K.C.V.O., F.R.C.S. Pp. 29. London: Longmans, Green, & Co. 1905.—This Lecture, reprinted from the *Lancet*, is based upon a collection of 750 cases of recurrent effusion in the knee-joint. The term recurrent effusion is held to mean an effusion which had recurred after an interval or intervals during which the joint, so far as could be seen, had resumed its normal condition. The cause of the first or original effusion in each case was an injury. Thus first effusions and effusions from tubercular disease are excluded from consideration. More than half the cases—428 to be precise—are classified as examples of the so-called "internal derangement" of the knee-joint with obvious signs. The diagnosis in this group of cases was verified by operation 80 times, but in the remaining 348 cases it was based upon the obvious signs alluded to above. Seven types of internal derangement were found in the 80 cases operated upon, and drawings illustrating these are appended. In another group of 56 cases, without signs of any kind excepting recurrent effusion, 12 were submitted to operation. In 7 of these the semilunar cartilages were found to be displaced, but in the remaining 5 nothing was found to account for the effusion. The technique of the operation for internal derangement of the knee-joint is described. In 241 other cases of recurrent effusion certain constitutional causes were influential, including malaria and some unknown cause which leads to passive or quiet effusion in certain young people, as well as the more ordinary conditions such as gout, rheumatism, and osteo-arthritis. The lecture is of considerable interest, and a recollection of the main points brought to notice cannot fail to be helpful to practitioners confronted with cases of recurring effusion in the knee-joint.

**The Ocular Circulation.** By J. HERBERT PARSONS, B.S., B.Sc., F.R.C.S. Pp. 76. London: John Bale, Sons and Danielsson, Ltd. 1903.—The author first points out the difference between the ocular blood supply in man and in the higher vertebrates, the latter having in most cases branches from the external carotid supplying the eyeball. In the lower vertebrates, and even in most of the lower mammals, the retina is entirely devoid of vessels, whilst in the elephant, horse, rodents, &c., the retinal vessels are extremely inconspicuous. Details of various experiments on the intra-ocular blood-supply and tension in animals are related, and manometer tracings are given which show that the eyeball, whilst having the highest capillary and venous pressure of any organ in the body, depends for its tension much more upon the general blood pressure than on vaso-dilator or vaso-constrictor influences of the trigeminal or sympathetic. There is partial analogy between intra-cranial and intra-ocular circulation, and increased intra-cranial pressure causes a rise in the tension of the eyeball. He suggests a new, and we think most probable, theory to account for optic neuritis. He compares it to the well-known œdema which surrounds a cerebral tumour, the œdema being in both instances due to increased pressure and consequent malnutrition. "A very slight œdema of the nerve at the lamina cribrosa will compress the capillaries, lead to greater anæmia and greater œdema, until finally the larger vessels are also compressed." In the last chapter some pathological anomalies of the ocular circulation are considered, the conditions existing in glaucoma, embolism of the central artery, and toxic amblyopias. Tobacco amblyopia, he finds, is due to a toxic effect upon the retinal elements, whilst quinine amblyopia is due to extreme constriction of the retinal arteries.

**A Manual of Ophthalmic Practice.** By CHARLES HIGGENS. Second Edition. Revised by ARTHUR W. ORMOND. Pp. viii., 375. London: H. K. Lewis. 1903.—The author says that he does not pretend to go deeply into the subject, and we quite agree with him. As far as the descriptions go there is nothing to complain of, but the whole is too superficial for the practitioner. It might do as a cram for students going up for a surgical examination, but it would not be of much use to anyone consulting it with a view to the treatment of obscure and difficult cases. The treatment of ophthalmia is fairly good, but no mention is made of the difficulty of cleansing a baby's eyes. Under episcleritis there is no mention of salicylate of sodium. The second half of the book deals with operations, and is more satisfactory. It is evidently written as the result of wide personal experience. We can recommend the book to anyone wishing a good general idea of the subject; it is up to date and accurate, and the treatment when sufficiently detailed is on modern lines and thoroughly reliable.

**Errors of Refraction and their Correction.** By Harold B. Grimsdale, M.B., B.C. Pp. iv., 87. London: The Medical Times Limited. 1904.—Another book on *Errors of Refraction and their Correction* is born to the world: this time Mr. Harold Grimsdale is the father. The book is carefully compiled and covers the whole subject. The subject, however, is limited, has been covered many times before, and by very capable hands. It seems almost unnecessary to do it again; still, this book goes into the matter in such a practical manner, that its study will doubtless be found a useful one.

**Guide to the Examination of the Throat, Nose and Ear, for Senior Students and Junior Practitioners.** By William Lamb, M.D., C.M. Edin. Pp. xi., 152. London: Baillière, Tindall & Cox. 1904.—To the class of readers mentioned in the title this little book may be cordially recommended. The best section of it, in our view, is that devoted to the description of the nasal cavities, and the value of this section is much enhanced by the excellent anatomical illustrations it contains. But good as this portion of the book is, it might well have been extended to include a more detailed account of how attempts should be made to discover the source of nasal suppuration originating in the accessory nasal sinuses. The portions of the book dealing with the examination of the larynx and ear are good, and also the concluding section, in which the elementary principles of local treatment are discussed. The initial difficulties of students in the examination of the throat, nose and ear often lead to discouragement, and secondarily to neglect. The numerous opportunities afforded in hospital practice of making these examinations are allowed to slip by unheeded, and in most cases they can never again be obtained. We think that the perusal by students in their earlier period of study in hospitals of some such manual as the one before us, which is short and written interestingly and lucidly, would help to diminish the discouragements to which we have alluded and to smooth the way for the acquirement of the skill necessary for the useful examination of the organs under consideration.

**An Introduction to Dermatology.** By Norman Walker, M.D. Third Edition. Pp. xvi., 284. Bristol: John Wright & Co. 1904. That a third edition of this work should be called for in so short a time is a sure sign that it is appreciated by the medical profession. A good deal of argumentative matter has been omitted in this edition, thus enabling the author to introduce some new matter without increasing the size of the book. A large part of this extra space is taken up with an account of the rôle that electro-therapy plays in dermatology. The author speaks very highly of the use of X-rays in certain skin complaints. Thus in speaking of the treatment of sycosis, he says: "The X-rays are probably more useful than any other treatment in obstinate cases." Again, speaking of the use of the rays in the treatment of favus, he says: "Their introduction has entirely altered the formerly gloomy prognosis of this disease." This hand-book of dermatology is to be thoroughly recommended both to the student and to all those who are interested in the subject.

**Practical Manual of Diseases of Women and Uterine Therapeutics.** By H. MACNAUGHTON-JONES, M.D., M.Ch. Ninth Edition. Pp. xxxviii., 1044. London: Baillière, Tindall & Cox. 1904.—It is now twenty years since the first edition of this work appeared. It has now assumed a very large size. It is too big for students, but for those engaged especially in the study of gynæcology it will be found very useful. One often wishes an author would give the methods he himself prefers, and not merely tabulate a number from other works. In this book not only does the author give his own ideas, but also those of others, not merely from reading or hearsay, but by having actually visited and seen their work in their respective clinics. In this way the book may be regarded as one of the best, in giving a broad, unbiased view of the whole subject, many descriptions and plates appearing in it for the first time in any book in this country. It is impossible to review the whole in detail. Perhaps the most prominent feature is the care with which the steps of all the operations are described and illustrated. Some of the smaller subjects—for instance, vascular caruncle—are not so well dealt with. In this edition the whole book has been profoundly altered and very much improved.

**Burdett's Hospitals and Charities, 1905.** By Sir HENRY BURDETT, K.C.B. London: The Scientific Press, Limited.—This year-book of philanthropy and the hospital annual is for the first time able to give complete figures for ten consecutive years during which the use of the uniform system of registration has been enforced throughout the hospitals of the metropolis. Fourteen preliminary chapters give the newest information and an exhaustive review of the more important questions in hospital administration which are engaging the attention of those interested in this important subject. Everything can here be found, from the duties of the chairman and medical superintendent to the most minute detail of income and expenditure. It will be a most fascinating book to those who revel in figures and in dictionaries. Its compilation has entailed vast labour, but great care has been exercised, and it is quite up to date. The name of its editor is a sufficient guarantee that no pains have been spared to make it as useful as is possible.

